# The climatic factors affecting dengue fever outbreaks in southern Taiwan: an application of symbolic data analysis

**DOI:** 10.1186/s12938-018-0575-4

**Published:** 2018-11-06

**Authors:** Yi-Horng Lai

**Affiliations:** Department of Health Care Administration, Oriental Institute of Technology, No. 58, Sec. 2, Sichuan Rd., Banqiao Dist., New Taipei City, 22061 Taiwan

**Keywords:** Dengue fever, Climate factors, Interval-valued data, Symbolic data analysis

## Abstract

**Background:**

Dengue fever is a leading cause of severe illness and hospitalization in Taiwan. This study sought to elucidate the linkage between dengue fever incidence and climate factors.

**Results:**

The result indicated that temperature, accumulated rainfall, and sunshine play an important role in the transmission cycles of dengue fever. A predictive model equation plots dengue fever incidence versus temperature, rainfall, and sunshine, and it suggests that temperature, rainfall, and sunshine are significantly correlated with dengue fever incidence.

**Conclusions:**

The data suggests that climate factors are important determinants of dengue fever in southern Taiwan. Dengue fever viruses and the mosquito vectors are sensitive to their environment. Temperature, rainfall and sunshine have well-defined roles in the transmission cycle. This finding suggests that control of mosquito by climatic factor during high temperature seasons may be an important strategy for containing the burden of dengue fever.

## Background

Dengue Fever is a common epidemiological mosquito-borne disease in subtropical and tropical regions and has become one of the public health’s biggest challenges. Dengue is a febrile illness caused by one of the antigenically different serotypes of dengue viruses and mainly transmitted to human through the bite of vectors, including *Aedes aegypti* and *Aedes albopictus* [1]. One area that has received particular attention is the association between climatic factors and vector-borne diseases [2].

Among the 193 WHO member countries, more than 70% of the populations are at risk of dengue. WHO reported that 390 million dengue infections occurred every year before 2013 [3]. The dengue infected regions include South-East Asia and Western Pacific, with the most vulnerable area in developing countries. The outbreaks do not only occur in rural areas but also in urban areas.

Taiwan is located in the Pacific Ocean region and is a hotbed of dengue vectors because of its high temperature and humidity [4]. The risk of dengue fever has increased gradually in southern Taiwan and has become a major public health issue that affects the quality of life and the health of Taiwan’s residents. During the first half of the twentieth century, there were three dengue fever outbreaks in Taiwan (1915, 1931, and 1942). After almost 40 years of dormancy, a dengue fever outbreak reoccurred in 2002 in southern Taiwan. The total number of indigenous cases in this outbreak was 5336, including 241 cases of dengue hemorrhagic fever (DHF) that caused 19 deaths. After that, the indigenous dengue cases were less than 400 in 2003–2005. Since 2006, Taiwan has faced dengue fever outbreaks of different scales every year; the cases were concentrated mainly in southern Taiwan, including Kaohsiung City, Tainan City, and Pingtung County. In 2015, Taiwan battled one of the most severe dengue outbreaks in history with over 42,000 dengue cases—22,741 cases in Tainan City, 18,933 cases in Kaohsiung City, and 373 cases in Pingtung City—and 228 deaths found to be associated with dengue infection [5].

Previous studies have been carried out on the correlation between climate factors and dengue fever using a wide spectrum of mathematical and statistical modeling methods [6–10]. Findings from most previous studies in other parts of the world also showed that climatic variables have an effect on dengue fever transmission. Studies in Taiwan [6, 11–13], Singapore [14], Vietnam [8, 15], Thailand [7, 16], China [9, 17], Trinidad [18, 19], Malaysia [20], Puerto Rico [21], Cambodia [10], and Saudi Arabia [22] showed a significant correlation between dengue fever incidence and temperatures, precipitation, and sunshine.

As temperature increases, the *Aedes aegypti* mosquito displays shorter periods of development in all stages of their life cycle, which leads to increased population growth. The mosquito feeding rate also increases; and dengue fever viruses in adult *Aedes aegypti* mosquitoes require shorter incubation periods to migrate to salivary glands [6–10, 14, 23]. Specifically, increasing temperatures increases the available habitat for the dengue fever vector, the *Aedes aegypti* mosquito, while concurrently increasing both the longevity of the virus and the mosquito [14]. Higher temperatures can also shorten the duration of virus replication, and increase mosquito reproduction and contacts with humans [9]. If temperature increases by approximately 3 °C, mean incidence rates during epidemics can double [24]. Warmer temperatures can increase the transmission rates of dengue fever in various ways. It may allow vectors to survive and reach maturity much faster than at lower temperatures [25]. Moreover, it may also reduce the size of mosquito larvae resulting in smaller adults that have high metabolism rates, requiring more frequent blood meal and need to lay eggs more often [25].

Some studies reported that rainfall can lead to increases in dengue fever transmission. They suggested that rainfall creates abundant outdoor breeding sources for *Aedes aegypti*, and the water storage containers also can serve as breeding habitats. Bhatt, Gething, Brady, Messina, Farlow, Moyes, Drake, Brownstein, Hoen, Sankoh, Myers, George, Jaenisch, Wint, Simmons, Scott, Farrar, and Hay paired the resulting risk map with detailed longitudinal information from dengue fever cohort studies, and they predicted dengue fever to be ubiquitous throughout the tropics, with local spatial variations influenced strongly by rainfall [23]. Choi, Tang, McIver, Hashizume, Chan, Abeyasinghe, Iddings, and Huy developed negative binomial models using monthly average maximum, minimum, mean temperatures and monthly cumulative rainfall, and they also claimed that rainfall significantly increased the dengue fever incidence [10]. When more consecutive wet days occurred in a period, dengue fever incidence increased. Rainfall leads to an increase in breeding sites of the mosquito vector, which would contribute to the increase in dengue fever occurrence [21]. On the contrary, however, some other studies showed that heavy rainfall can possibly lower dengue fever transmission by reducing the survival rate of the *Aedes aegypti* mosquito. Wegbreit analyzed weekly dengue fever morbidity data from the twin-island country of Trinidad and Tobago, and he suggested that there is a slightly negative correlation with the precipitation [26]. Thammapalo, Chongsuwiwatwong, McNeil, and Geater determine the independent effects of rainfall [16] in Thailand, and they also found that increased rainfall is associated with a decreased incidence of dengue fever cases in some provinces. Alshehri [22] aimed to address the effects of heavy rainfall on *Aedes aegypti* mosquito density in Saudi Arabia, and he argued that dengue fever has negative correlation with rainfall and humidity.

Sunshine is also closely linked to other ecological factors such as temperature and humidity and thereby might affect the dengue fever incidence [15]. Correlation studies carried out on monthly dengue fever cases have found the risk of dengue fever to be inversely associated with duration of sunshine [8]. With the monthly data in Vietnam, Vu, Okumura, Hashizume, Tran, and Yamamoto indicated that there is a significant negative association between dengue fever cases and the hours of sunshine [15]. Wongkoon, Jaroensutasinee, Jaroensutasinee investigated the effect of seasonal variation on the abundance of *Aedes aegypti* mosquito larvae and explored the impact of weather variability on dengue fever transmission in Thailand, and they concluded that maximum temperature, sunshine and evaporation are negatively correlated with dengue fever incidence [19].

However, while most studies claimed that climate is a determinant of dengue fever, some other studies argued that climate factor has no obvious correlation with this disease. They suggested that temperature [18, 27–29] and rainfall [13, 27–29] did not affect dengue fever incidence. The weekly average maximum temperature, total rainfall and the total number of dengue fever cases from 2005 to 2011 were used as time series data in Goto, Kumarendran, Mettananda, Gunasekara, Fujii, and Kaneko’s study [27]. They found that weekly average maximum temperatures and the weekly total rainfall did not significantly affect dengue fever incidence in three geographically different areas of Sri Lanka. Pandey, Nagar, Gupta, Khan, Singh, Mishra, Prakash, Singh, Singh, and Jain reported the annual trend of dengue fever virus infection in north India [28], and they indicated that there is no statistical significant correlation between weather data and increasing dengue fever positive cases. In a population-based study on the effects of climate and mosquito indices on dengue fever in Trinidad, Chadee, Shivnauth, Rawlins, and Chen declared that no significant correlations are observed between temperature and dengue fever [18]. Chang, Lee, Ko, Tsai, Lin, Chen, Lu, and Chen pointed out that climatic factors correlated significantly with case numbers of many diseases, such as murine typhus and Q fever, but neither temperature nor rainfall correlated with the case number of dengue fever [13]. According to the epidemiological investigation, the incidence of dengue fever had no relationship with temperature, or precipitation, and some studies [29] showed a clear relationship only with the sociological factors.

Most of the climatic data are range-type data. Because of the limitation of traditional statistics (e.g., regression analysis), range-type data is difficult to be analyzed. Most of them are analyzed by minimum value (e.g., minimum temperature), maximum value (e.g., maximum temperature), mean value (e.g., mean temperature, mean rainfall), and cumulative value (e.g., cumulative rainfall, cumulative sunshine). Most studies developed linear regression model [6–10] or negative binomial regression model [10] using monthly average temperatures [14, 17], maximum temperatures [7, 27], minimum temperatures [10], mean rainfall [14, 17], cumulative rainfall [27], and cumulative sunshine [8, 15, 19] over the period for the relationship between dengue fever incidence and climatic data. However, the major drawback of the traditional statistical methods is that when the correlation between dengue fever and each of the above-mentioned value is not consistent, it will be difficult to draw a conclusion [10, 19]. For example, in Choi, Tang, McIver, Hashizume, Chan, Abeyasinghe, Iddings, and Huy’s study [10], mean temperature is significantly associated with dengue fever incidence, but dengue fever incidence did not correlate well with the maximum temperature and minimum temperature. Wongkoon, Jaroensutasinee, Jaroensutasinee’s study also had the same problem. They investigated the effect of seasonal variation on the abundance of the *Aedes aegypti* mosquito larvae and explored the impact of weather variability on dengue fever transmission in Thailand, and they found that mean temperature and minimum temperatures are positively associated with dengue fever incidence, but maximum temperature is negatively correlated with dengue fever incidence [19].

With the advent of information technology, very large datasets have become routine. Traditional statistical methods do not have the power or flexibility to analyze these efficiently and extract the required knowledge. Symbolic data analysis is to summarize a large dataset in such a way that the resulting summary dataset is of a manageable size and yet retains as much of the knowledge in the original dataset as possible [30, 31]. One consequence of this is that the data may no longer be formatted as single values, but be represented by lists, intervals, distributions, etc. The summarized data have their own internal structure, which must be taken into account in any analysis.

High peaks for dengue outbreak is reported on summer in Taiwan. This suggests that climatic factors are likely to exert potential impact on dengue fever outbreak in tropical or subtropical regions [32, 33]. This study is aimed for investigate the relationship between climatic factors and the outbreaks of dengue fever in southern Taiwan with symbolic data analysis and to compare the differential effects of climatic factors on the incidence of dengue fever in southern Taiwan.

## Materials and research method

Climatic factors, such as temperature, rainfall, and sunshine play an important role in the spread of dengue fever viruses. The dengue fever data and climatic data of Kaohsiung city from January 2005 to March 2014 were analyzed with symbolic data analysis for the interval-valued data in this study.

### Study area

Kaohsiung City is the largest metropolitan city of southern Taiwan with an estimated population of 2,777,784 in 2016. According to the computerized database from the surveillance system of Taiwan’s Center for Disease Control, Kaohsiung City had 5543 confirmed dengue fever cases from January 2005 to March 2014, accounting for most of the total cases in Taiwan. Dengue fever transmission has been active in this area, and the latest large-scale outbreak occurred in the end of 2014.

### Data collection

Since 1988, dengue fever has been announced as a Class III Notified Disease in Taiwan, and the data are collected continuously and systematically by Taiwan’s Ministry of Health and Welfare with the Taiwan National Infectious Disease Statistics System [34]. The data collection mechanism has been stable over time, and this routinely-collected data can be used for analyzing factors affecting the occurrence of dengue fever. Because the most recent data is subject to update, this study focuses only on the data from January 2005 to March 2014.

Meteorological data on the monthly maximum temperature, minimum temperature, amount of rainfall, and amount of sunshine were obtained from the Climate Statistics Database provided by Taiwan’s Central Weather Bureau [35].

The variables that correlate with dengue fever are then submitted to symbolic linear regression analysis. Symbolic data analysis is employed to explore and identify statistically significant risk indicators [33].

### Symbolic data analysis

Symbolic data analysis is a relatively new field that provides a range of methods for analyzing complex datasets. Traditional statistical methods do not have the power or flexibility to make sense of very large datasets, and symbolic data analysis techniques can be developed in order to extract knowledge from such data. The analysis of symbolic data differs from that of the traditional. Rather than identifying points of interest in the data, symbolic data methods allow the user to build models of the data and make predictions about future events [32].

Dengue fever data of Kaohsiung city from January 2005 to August 2014 were analyzed with symbolic linear regression analysis for interval-valued data with R 3.3.2 (with Package RSDA) with SparkR 2.1 in symbolic data analysis with center-method [36, 37]. The regression model equation plots dengue fever incidence (cases) versus temperature (°C), accumulated rainfall (mm), and accumulated sunshine (hours) is as follows:

1 $$ {\text{Dengue fever incidence}} = \beta_{0} + \beta_{ 1} \times {\text{temperature}} + \beta_{ 2} \times {\text{accumulated rainfall}} + \beta_{ 3} \times {\text{accumulated sunshine}} $$

Billard and Diday proposed an approach for a constrained linear regression model on the midpoints and range of the interval values [30]. The prediction of the lower and upper boundaries of the interval value of the dependent variable is accomplished from its midpoint and range, which are estimated from the fitted linear regression models applied to the midpoint and range of each interval value of the independent variables.

Based on Billard and Diday’s study [30], the estimate of the parameters β is based only on the midpoint of the intervals according to the criterion considered. Let E = {e_1_,…,e_n_} be a set of examples that are described by p + 1 interval-valued variables y, x_1_,…,x_p_. Each example is represented as an interval quantitative feature vector z_i_ = (x_i_, y_i_), xi = (x_i1_,…,x_ip_), $x_{ij} = [a_{ij} ,b_{ij} ] \in \hat{s} = \{ [a,]:a,b \in R,a \le b\}$ where (j = 1,…,p) and $y_{i} = [y_{Li,} y_{Ui} ] \in \hat{s}$ are, respectively, the observed values of x_j_ and y.

It can be considered that X_1_,…,X_p_ related to Y according to the linear regression relationship:

$$ y_{Li} = \beta_{0} + \beta_{1} a_{i1} + \cdots + \beta_{p} a_{ip} + \varepsilon_{Li} $$

2 $$ y_{Ui} = \beta_{0} + \beta_{1} b_{i1} + \cdots + \beta_{p} b_{ip} + \varepsilon_{Ui} $$

From Eq. (2), the sum of the squares of deviations in this first approach is as follows:

3 $$ S_{cm} = \sum\limits_{i = 1}^{n} {( \in_{Li} + \in_{Ui} )^{2} } = \sum\limits_{i = 1}^{n} {(y_{Li} - \beta_{0} - \beta_{1} \alpha_{i1} } - \cdots \beta_{p} \alpha_{ip} + y_{Ui} - \beta_{0} - \beta_{1} b_{i1} - \cdots \beta_{p} b_{ip} )^{2} $$

which represents the sum of the square of the sum of the lower and upper boundary errors.

Lima Neto and de Carvalho present the estimates of the vector of parameters β in matrix notation for the center method [37], which can be rewritten in the simplest form as

4 $$ y^{C} = X^{C} \beta + \varepsilon^{C} $$

where $$ y^{C} = (y_{1}^{C} , \ldots y_{n}^{C} )^{T},$$ $$ X^{C} = (x_{1}^{C} )^{T} , \ldots (x_{n}^{C} )^{T} )^{T}, $$ $$ (x_{i}^{C} )^{T} = (1,x_{i1}^{C} , \ldots x_{ip}^{C} ) $$ $$ i = 1, \ldots ,n $$$$ \beta = (\beta_{0} , \ldots ,\beta_{p} )^{T} $$, and $$ \varepsilon^{C} = (\varepsilon_{1}^{C} , \ldots ,\varepsilon_{n}^{C} )^{T}. $$

If X_c_ has full rank (p + 1) ≦ n, the least square estimate of β in Eq. (3) is given by

5 $$ \hat{\beta } = ((X^{C} )^{T} X^{C} )^{ - 1} (X^{C} )^{T} y^{C} $$

Given a new example e, described by z = (x, y), where x = (x_1_,…,x_p_) with x_j_ = [a_i_, b_j_] (j = 1,…,p), the value y = [y_L_, y_U_] of Y will be predicted by $\hat{y} = [\hat{y}_{L} ,\hat{y}_{U} ]$ as follows:

6 $$ \hat{y}_{L} = (x_{L} )^{T} \hat{\beta }\,{\text{and}}\,\hat{y}_{U} = (x_{U} )^{T} \hat{\beta } $$

where $$ (x_{L} )^{T} = (1,a_{1} , \ldots ,a_{p} ) $$,

$$ (x_{U} )^{T} = (1,b_{1} , \ldots ,b_{p} ). $$

The determination coefficient (R^2^) represents a goodness-of-fit measure commonly used in regression analysis to capture the adjustment quality of a model. The determination coefficient (R^2^) for the CM method is easily established as

7 $$ R_{cm}^{2} = \frac{{\sum\nolimits_{i = 1}^{n} {(\hat{y}_{i}^{C} - \bar{y}^{C} )^{2} } }}{{\sum\nolimits_{i = 1}^{n} {(y_{i}^{C} - \bar{y}^{C} )^{2} } }} $$

However, note that $y^{C} = (y_{L} + y_{U} )$. Thus, the Eq. (7) can be replaced by

8 $$ R_{cm}^{2} = \frac{{\sum\nolimits_{i = 1}^{n} {((\hat{y}_{Li} + \hat{y}_{Ui} ) - (\bar{y}_{Li} + \bar{y}_{Ui} ))^{2} } }}{{\sum\nolimits_{i = 1}^{n} {((y_{Li} + y_{Ui} ) - (\bar{y}_{Li} + \bar{y}_{Ui} ))^{2} } }} $$

Billard and Diday’s method [30] indicate the importance of range-type information in prediction performance as well as the application of inequality constraints to ensure mathematical coherence between the predicted values of the lower and upper boundaries of the interval-value data.

## Results

Using monthly aggregated data, dengue fever data of Kaohsiung city from January 2005 to March 2014 were analyzed with symbolic linear regression analysis for the interval-valued data in this study. The climatic data in this study includes temperature, rainfall, and sunshine. The type of these data is interval-valued data, and they all can be presented as U[lower, upper], meaning the range of the data is from minimum (lower) to maximum (upper). Details of the dengue fever incidence in Kaohsiung City and the monthly temperature/rainfall/sunshine measurements are presented in Tables 1 and 2 respectively.

**Table 1:** The characteristics of the research data in dengue fever data

	N (months)	Total (years)	Mean (months)	S. E. (months)
2005/Jan.–2005/Dec.	12	144	12.00	15.82
2006/Jan.–2006/Dec.	12	956	79.67	100.36
2007/Jan.–2007/Dec.	12	202	16.83	20.60
2008/Jan.–2008/Dec.	12	443	36.92	48.67
2009/Jan.–2009/Dec.	12	773	64.42	102.70
2010/Jan.–2010/Dec.	12	1106	92.17	122.07
2011/Jan.–2011/Dec.	12	1183	98.58	143.43
2012/Jan.–2012/Dec.	12	532	44.33	57.98
2013/Jan.–2013/Dec.	12	102	8.50	9.74
2014/Jan.–2014/Mar.	3	7	2.33	3.47

**Table 2:** The characteristics of the research data in climatic data

	N (months)	Temperature (°C)	Rainfall (mm)	Sunshine (hours)
		U[lower, upper]	U[lower, upper]	U[lower, upper]
2005/Jan.–2005/Dec.	12	U[8.7, 34.9]	U[0, 1030.0]	U[0, 259.4]
2006/Jan.–2006/Dec.	12	U[11.3, 35.7]	U[0, 901.5]	U[0, 224.6]
2007/Jan.–2007/Dec.	12	U[9.2, 35.4]	U[0, 1229.3]	U[0, 290.6]
2008/Jan.–2008/Dec.	12	U[10.2, 35.0]	U[0, 1199.7]	U[0, 240.1]
2009/Jan.–2009/Dec.	12	U[9.3, 35.0]	U[0, 934.5]	U[0, 256.0]
2010/Jan.–2010/Dec.	12	U[10.9, 35.9]	U[0, 853.0]	U[0, 238.6]
2011/Jan.–2011/Dec.	12	U[11.3, 35.8]	U[0, 543.0]	U[0, 240.9]
2012/Jan.–2012/Dec.	12	U[11.0, 36.4]	U[0, 832.5]	U[0, 231.8]
2013/Jan.–2013/Dec.	12	U[12.0, 35.7]	U[0, 765.7]	U[0, 248.1]
2014/Jan.–2014/Mar.	3	U[11.1, 30.6]	U[0, 67.0]	U[0, 254.8]

**Fig. 1:**
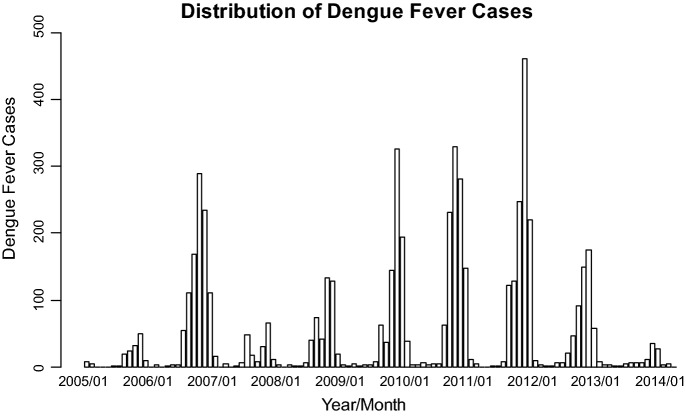
Distribution of dengue fever cases from January 2005 to March 2014

**Fig. 2:**
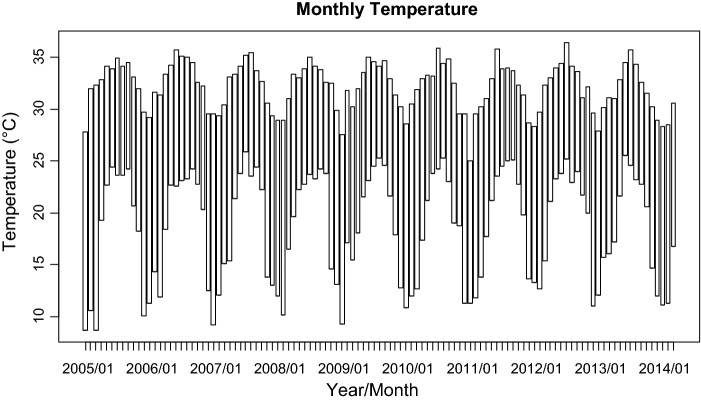
Distribution of temperature from January 2005 to March 2014

**Fig. 3:**
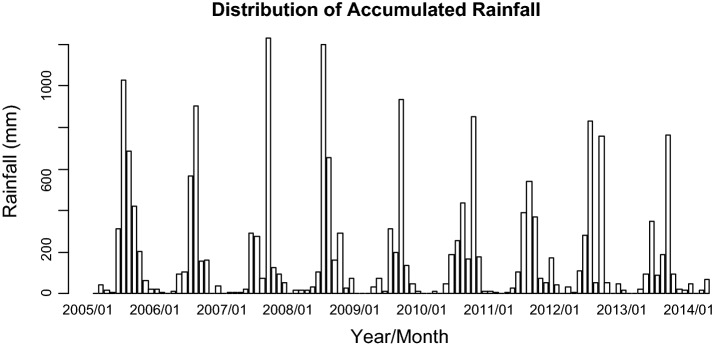
Distribution of accumulated rainfall from January 2005 to March 2014

**Fig. 4:**
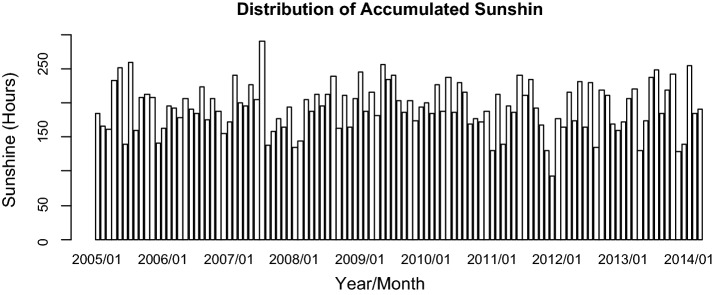
Distribution of accumulated sunshine from January 2005 to March 2014

For the 111-month period (January 2005–March 2014), the number of dengue fever cases in Kaohsiung City was from 0 to 462, with the highest one (462 cases) occurred in November 2011 (Fig. 1). The monthly minimum temperature was from 8.7 °C to 12.0 °C, with the lowest recorded minimum temperature in January 2005 and March 2005. The monthly maximum temperature was from 30.6 to 36.4 °C, with the highest recorded minimum temperature in July 2012 (Fig. 2). The monthly accumulated rainfall measurement was from 0 to 1229.3 mm, with the highest recorded measurement in August 2007 (Fig. 3). The monthly accumulated sunshine measurement was from 0 to 290.6 h, with the highest recorded measurement in July 2007 (Fig. 4).

The predictive model equation plots dengue fever incidence (cases) versus temperature (°C), accumulated rainfall (mm), and accumulated sunshine (hours) is as follows:

9 $$ {\text{Dengue fever incidence}}\,=\,3.118\,+\,6.289 \times {\text{temperature}}\,+\,\left({-\;0.144} \right) \times {\text{rainfall}}\,+\,\left( {- 1.025} \right) \times {\text{sunshine}}$$

**Fig. 5:**
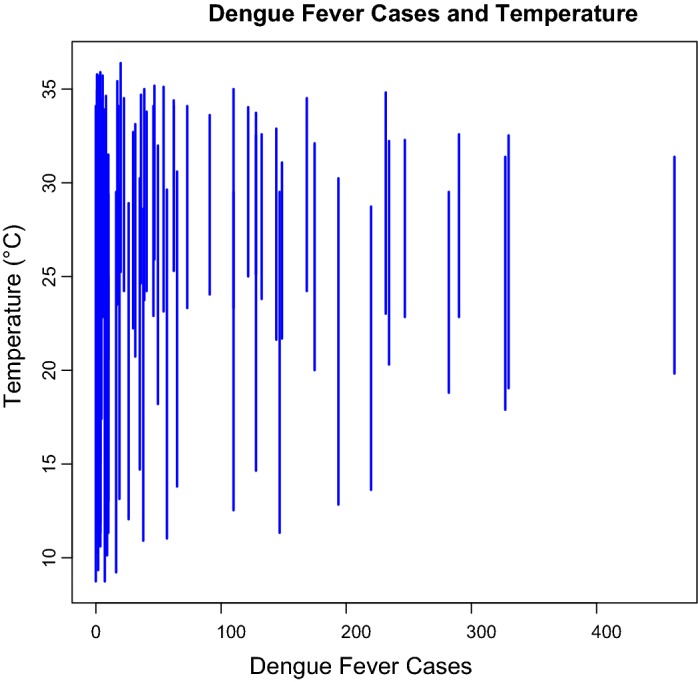
The symbolic scatter plot of dengue fever cases with accumulated temperature

**Fig. 6:**
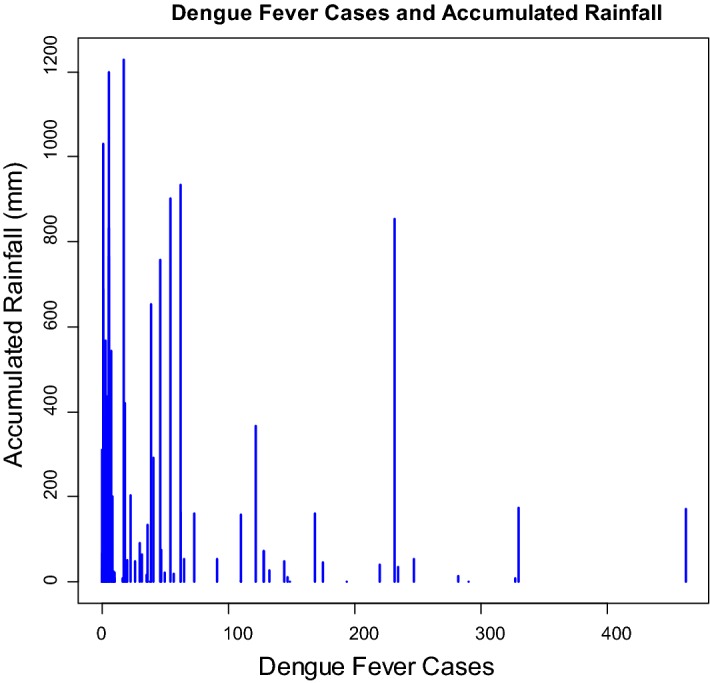
The symbolic scatter plot of dengue fever cases with accumulated rainfall

**Fig. 7:**
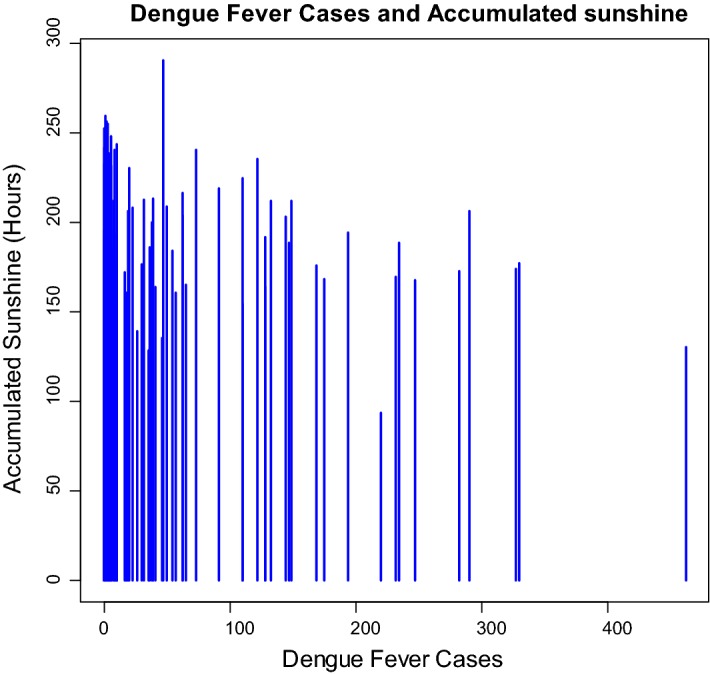
The symbolic scatter plot of dengue fever cases with accumulated sunshine

The results demonstrate that climatic factors are associated with dengue fever cases. Table 3 shows that, first, monthly temperature (t-value = 2.282, *p* value = 0.024) is positively correlated with the dengue fever cases, and this result is in agreement with previous studies [6–10, 14, 23]. Second, same with Wegbreit’s study [26] and Alshehri’s study [22], accumulated rainfall (t-value = − 2.002, p-value = 0.047) is negatively correlated with the dengue fever cases. Third, accumulated sunshine (t-value = − 2.790, p-value = 0.007) is also negatively correlated with the dengue fever cases, and this result supports Vu, Okumura, Hashizume, Tran, and Yamamoto’s research [15]. The symbolic scatter plot of dengue fever cases with temperature/accumulated rainfall/accumulated sunshine is presented in Figs. 5, 6, and 7. The r-squared value is .138, which indicates that 13.8% of the error is explained by the model.

**Table 3:** Regression analysis of climatic factor affecting dengue fever incidence in southern Taiwan

	Estimate	S. E.	t-value	p-value
Intercept	3.118	66.797	0.047	0.963
Temperature	6.289	2.756	2.282	0.024
Rainfall	− 0.144	0.072	− 2.002	0.047
Sunshine	− 1.025	0.368	− 2.790	0.007

## Discussion

From the study that has been carried out, it is possible to conclude that the risk of dengue fever is positively associated with high temperature, but inversely associated with duration of rainfall and sunshine. This result is consistent with findings of most of the previous studies.

This study shows that temperature is significantly associated with dengue fever incidence. As the temperature increases, the transmission rate of dengue fever also increases. The weight of temperature is larger than others in the predictive model, and it is possible that temperature plays an important role in most of the physiological functions of vectors in southern Taiwan. Temperature is an important climatic factor affecting biological processes of mosquitoes, including their interactions with viruses. Temperature is also positively associated with pre-adult mosquito maturation, oviposition rate, and virus incubation rate in mosquitos [11].

It is found in this study that rainfall and sunshine are both negatively associated with the transmission of dengue fever in southern Taiwan. Figure 2 shows that heavy rainfalls are frequent in southern Taiwan. According to previous studies [16, 22, 26], heavy rainfalls may not favor mosquito density as most of the mosquito eggs and larvae would be washed away from breeding sites [26]. This argument may explain why dengue fever decreases as rainfall increases in southern Taiwan.

Sunshine is also closely linked to other ecological factors such as temperature and thereby might affect the dengue fever incidence. The longer the hours of sunshine, the higher of the temperature is. Although warmer temperatures can increase the transmission rates of dengue fever in various ways, many studies have concluded that high temperature may not favor mosquito density as most of the mosquito larvae would die in heat [19]. This may be the reason why previous studies [10, 19, 20] indicated that maximum temperature and sunshine are negatively correlated with dengue fever incidence.

## Conclusions

Dengue fever is ubiquitous throughout the tropics. With spatial variation in different regions, the effects of climate on dengue fever are also different. Dengue viruses and their mosquito vectors are sensitive to their environment. Temperature, rainfall and sunshine have well-defined roles in the transmission cycle. Findings of this paper suggest that control of mosquito by climatic factor during high temperature seasons may be an important strategy for containing the burden of dengue fever.

This study only concentrated on climatic factors. However, some studies [29] showed a clear relationship between sociological factors (e.g., population, urbanization public health policy, and health education) and dengue fever. Further research can continue to test and verify those. In addition, this study focused on Taiwan. Taiwan is a region that is influenced deeply by Chinese culture, so this study can be seen as an example of how climatic factors affect dengue fever in Chinese lifestyle. Future research can further explore how lifestyles in different cultures can influence the occurrence of dengue fever.
